# RAB10 Interacts with the Male Germ Cell-Specific GTPase-Activating Protein during Mammalian Spermiogenesis

**DOI:** 10.3390/ijms18010097

**Published:** 2017-01-05

**Authors:** Ying-Hung Lin, Chih-Chun Ke, Ya-Yun Wang, Mei-Feng Chen, Tsung-Ming Chen, Wei-Chi Ku, Han-Sun Chiang, Chung-Hsin Yeh

**Affiliations:** 1 Graduate Institute of Biomedical and Pharmaceutical Science, Fu-Jen Catholic University, New Taipei City 24205, Taiwan; 084952@mail.fju.edu.tw (Y.-H.L.); 053824@mail.fju.edu.tw (H.-S.C.); 2 Department of Urology, En Chu Kong Hospital, New Taipei City 23702, Taiwan; koacurtis@gmail.com; 3 Department of Chemistry, Fu Jen Catholic University, New Taipei City 24205, Taiwan; vic0009@gmail.com; 4 Bone and Joint Research Center, Chang Gung Memorial Hospital, Taoyuan 33305, Taiwan; mfchen0@gmail.com; 5 Department and Graduate Institute of Aquaculture, National Kaohsiung Marine University, Kaohsiung 81157, Taiwan; tmtonychen@webmail.nkmu.edu.tw; 6 School of Medicine, Fu Jen Catholic University, New Taipei City 24205, Taiwan; 089052@mail.fju.edu.tw; 7 Division of Urology, Department of Surgery, Shin-Kong Wu-Su Memorial Hospital, Taipei 11101, Taiwan

**Keywords:** *Male Germ Cells Rab GTPase-Activating Proteins*, RAB10, spermatogenesis

## Abstract

According to recent estimates, 2%–15% of couples are sterile, and approximately half of the infertility cases are attributed to male reproductive factors. However, the reasons remain undefined in approximately 25% of male infertility cases, and most infertility cases exhibit spermatogenic defects. Numerous genes involved in spermatogenesis still remain unknown. We previously identified *Male Germ Cells Rab GTPase-Activating Proteins* (*MGCRABGAPs*) through cDNA microarray analysis of human testicular tissues with spermatogenic defects. MGCRABGAP contains a conserved RABGAP catalytic domain, TBC (Tre2/Bub2/Cdc16). RABGAP family proteins regulate cellular function (e.g., cytoskeletal remodeling, vesicular trafficking, and cell migration) by inactivating RAB proteins. MGCRABGAP is a male germ cell-specific protein expressed in elongating and elongated spermatids during mammalian spermiogenesis. The purpose of this study was to identify proteins that interact with MGCRABGAP during mammalian spermiogenesis using a proteomic approach. We found that MGCRABGAP exhibited GTPase-activating bioability, and several MGCRABGAP interactors, possible substrates (e.g., RAB10, RAB5C, and RAP1), were identified using co-immunoprecipitation (co-IP) and nano liquid chromatography-mass spectrometry/mass spectrometry (nano LC-MS/MS). We confirmed the binding ability between RAB10 and MGCRABGAP via co-IP. Additionally, MGCRABGAP–RAB10 complexes were specifically colocalized in the manchette structure, a critical structure for the formation of spermatid heads, and were slightly expressed at the midpiece of mature spermatozoa. Based on these results, we propose that MGCRABGAP is involved in mammalian spermiogenesis by modulating RAB10.

## 1. Introduction

### 1.1. Male Infertility

It is reported that approximately 2%–15% of couples worldwide are affected by infertility, of which nearly half are attributed to male reproductive factors [1,2]. Male infertility is a multifactorial syndrome encompassing a wide variety of disorders such as anatomic defects, gametogenesis dysfunction, endocrinopathies, immunological problems, ejaculatory failure, environmental exposures, and genetic mutations [3]. However, the cause is unknown in up to 25% of male infertility cases [4]. Genetic abnormalities leading to male infertility are responsible for 15%–30% of the cases, and it is likely that most cases of idiopathic infertility are constituted by underlying genetic causes [5]. Molecular defects and genetic alterations responsible for male infertility disrupt physiological processes, including hormonal homeostasis, spermatogenesis, and sperm quality [6]. However, a high number of genes involved in spermatogenesis still remain unknown [7].

### 1.2. Male Fertility

The landscape of male reproduction is composed of spermatogenesis, spermiogenesis, maturation of spermatozoa in the epididymis, capacitation, and fertilization. In the seminiferous tubules of the testis, spermatogenesis is the continuing mitotic proliferation of the spermatogonia and meiotic division of spermatocytes [8]. Furthermore, there are a series of morphological changes to produce a haploid gamete during spermiogenesis [8,9]. Furthermore, spermatozoa transport to the epididymis for the further maturation of the spermatozoa, including their acquisition of progressive motility and fertilizing ability [10]. After ejection, the spermatozoa move into the female reproductive tract. During capacitation, the chemicals of the female reproductive environment trigger a series of changes in the cellular physiology of the spermatozoa (e.g., intracellular calcium concentration, cell membrane fluidity, and kinase activity) [11,12]. Finally, several steps of fertilization include spermatozoa-oocyte binding, acrosomal reaction, egg cortical reaction, and sperm-egg fusion. This complex process is highly ordered and requires the precise coordination of cell cycle events as well as regulatory pathways, which depend on the expression of many genes. These genes and proteins are male fertility biomarkers [13,14].

### 1.3. Identification of MGCRABGAP as a Novel Sterile-Related Gene

We previously identified novel testis-specific genes through cDNA microarray analysis of human testicular tissues [15]. From this analysis, we identified the human *Male Germ Cells Rab GTPase-Activating Proteins* (*MGCRABGAPs*), characterized by the presence of a conserved RABGAP catalytic domain, TBC (Tre2/Bub2/Cdc16) [16]. Proteins containing TBC domains are largely conserved in eukaryotic organisms and function as GTPase-activating proteins (GAPs) for Rab GTPases [17]. The expression of MGCRABGAP is abundant in the peri-acrosomal and manchette regions of elongating and elongated spermatids and the tail region of mature spermatozoa. MGCRABGAP has been suggested to be a key element in acrosome and manchette formation and sperm-tail reorganization during mammalian spermiogenesis [16]. From these results, MGCRABGAP may be involved in forming the acrosome, shaping the sperm head, and elongating the sperm tail by modulating its potential substrates, RABs. 

### 1.4. RABs and Their Regulators

RAB proteins are small GTP-binding proteins of approximately 20–40 kDa that form the largest subfamily within the RAS GTPase superfamily; these proteins are key regulators of membrane trafficking and fusion events [18]. RABs function as “molecular switches” by shuttling between GTP-bound active forms and GDP-bound inactive forms. GDP-GTP cycling is regulated by guanine nucleotide exchange factors and GAPs, which catalyze the activation and deactivation of GTPases, respectively. In addition to their function as regulators of membrane traffic, RAB GTPases have been shown to participate in cell proliferation, innate immune responses, apoptosis, and nuclear signaling [19,20].

### 1.5. RABs and Male Ferttility 

Several RABs (e.g., RAB2A, RAB3A, RAB5, RAB6, RAB7, RAB8B, RAB13, and RAB27A/B) have been implicated in mammalian spermatogenesis [21,22,23,24,25,26,27,28,29,30]. RAB2A, RAB3A, RAB6, RAB7, and RAB27A/B are expressed at the acrosome and/or manchette, which are involved in sperm head and acrosomal function. Furthermore, RAB5, RAB8B, and RAB13 contribute to intracellular trafficking at the adherens junction of the testis. In addition, RAB2, RAB3, and RAB27 are involved in acrosomal reaction and fertilization through facilitating exocytosis or modifying acrosome structure [11,31].

In this study, we identified possible interacting proteins of MGCRABGAP to reveal the possible roles of MGCRABGAP during mammalian spermiogenesis. 

## 2. Results

### 2.1. Evaluation of the GTPase-Activating Bioability of MGCRABGAP in NTERA-2 cl.D1 Cells

**Figure 1:**
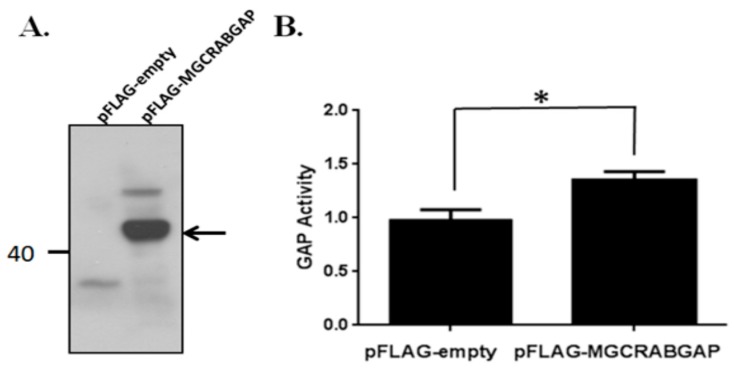
Male Germ Cells Rab GTPase-Activating Proteins (MGCRABGAP) expressed GTPase-activating activity in NT2D1 cells; (**A**) The total cell lysates of NT2D1 cells transfected with the empty pFLAG or pFLAG-MGCRAB vector were immunoblotted; Blots were probed with an anti-FLAG antibody; The arrow points to MGCRABGAP; (**B**) The lysates were used to evaluate GTPase-activating bioability (*n* = 4); Statistical significance (* *p* < 0.05) was determined via paired *t*-test methods; NT2D1: NTERA-2 cl.D1cell line; GAP: GTPase-Activating Protein.

To determine the GTPase-activating bioactivity of MGCRABGAP, MGCRABGAP overexpression was induced, and a GTPase-activating assay was performed. Human MGCRABGAP was cloned into the pFLAG vector and transfected into the NTERA-2 cl.D1cell line (NT2D1), a pluripotent human testicular embryonal carcinoma cell line. Figure 1 indicates that the GTPase-activating bioactivity of MGCRABGAP was significantly enhanced in comparison with the cells transfected with an empty vector. 

### 2.2. Searching for MGCRABGAP Substrates

**Figure 2:**
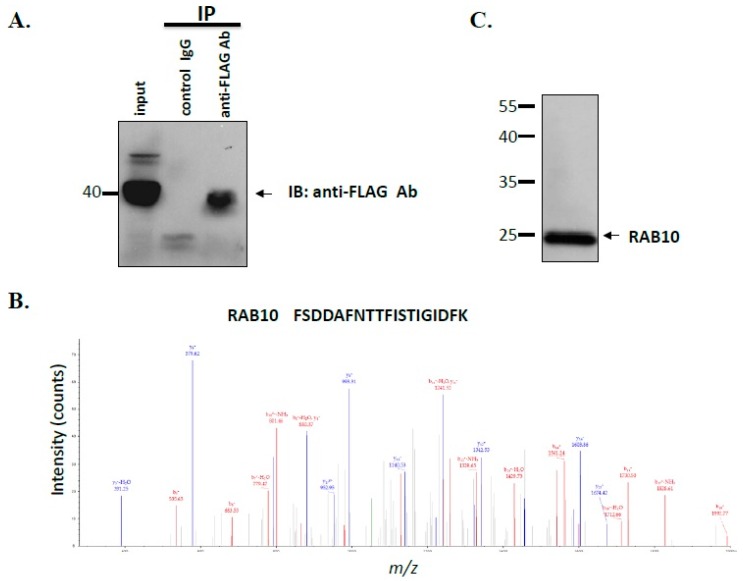
Identification of interactors of MGCRABGAP through co-immunoprecipitation (co-IP) and nano LC-MS/MS; (**A**) Coimmunoprecipitation assay; Lysates from the cells transfected with the pFLAG-MGCRABGAP plasmid were immunoprecipitated with an anti-FLAG antibody (anti-FLAG Ab) or a nonspecific immunoglobulin G (IgG) control (control IgG), followed by immunoblotting with the anti-FLAG antibody; Protein input (5%) served as an immunoblotting control (input); The arrow points to MGCRABGAP; (**B**) MS/MS spectrum of the tryptic peptide FSDDAFNTTFISTIGIDFK for RAB10; (**C**) Immunoblotting of NT2D1 lysates with the anti-RAB10 antibody; Ab: antibody.

For identification of interacting proteins of MGCRABGAP, coimmunoprecipitation (co-IP) and nano liquid chromatography-mass spectrometry/mass spectrometry (nano LC-MS/MS) were performed. After the cells were transfected with the pFLAG-MGCRABGAP plasmid, the lysates were co-immunoprecipitated with mouse immunoglobulin G (IgG) and the anti-FLAG antibody. The specific binding of co-IP was evaluated through immunoblotting with the anti-FLAG antibody (Figure 2A). As shown in Table 1, several possible interactors of MGCRABGAP were identified, namely RAB10, RAB5C, RAB1A, RAP1A, and RAP1B. Figure 2B,C show the results of nano LC-MS/MS and immunoblotting of RAB10, respectively. Through proteomic tools, we identified several possible interactors of MGCRABGAP. 

### 2.3. RAB10 Interacted and Colocalized with MGCRABGAP in Male Germ Cells

**Figure 3:**
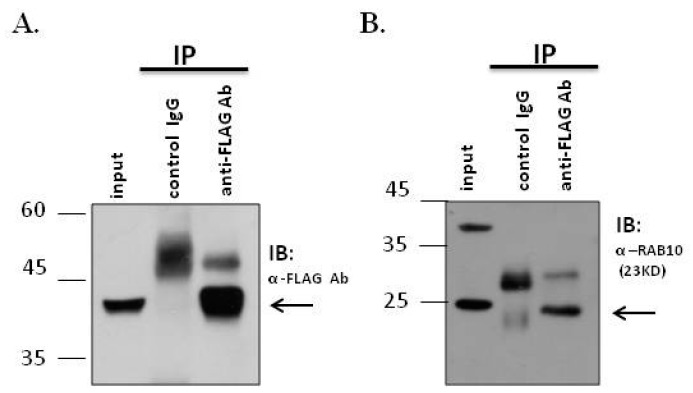
MGCRABGAP interacts with RAB10 in a male germ cell line; Co-IP of FLAG-MGCRABGAP with RAB10; (**A**) and (**B**) Lysates from cells transfected with pFLAG-MGCRABGAP and then immunoprecipitated with an anti-FLAG antibody (anti-FLAG Ab) or a nonspecific IgG control (control IgG), followed by immunoblotting with the anti-FLAG antibody (**A**) or anti-RAB10 antibody (**B**); Input protein (5%) was used as an immunoblotting control (input).

**Figure 4:**
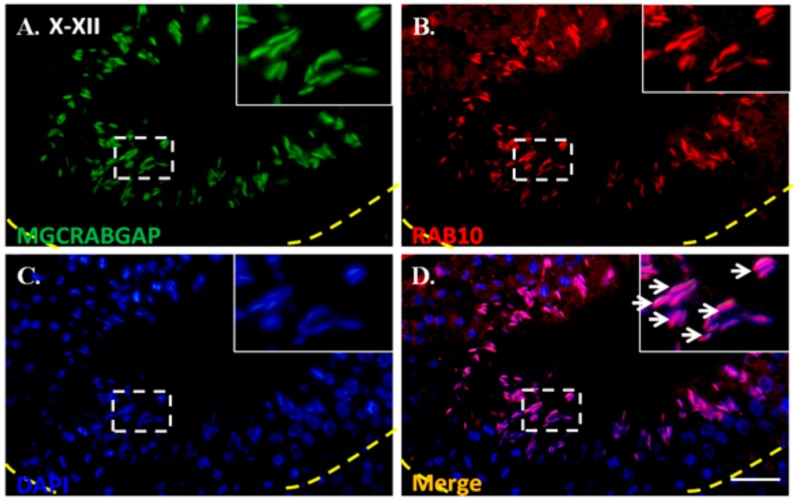
Colocalization of MGCRABGAP and RAB10 in murine testicular sections; Colocalization of MGCRABGAP with RAB10 in adult murine testicular sections; (**A**–**D**), anti-MGCRABGAP antibody (green); anti-RAB10 antibody (red); 4′,6-diamidino–2-phenylindole (DAPI) (blue); merging of MGCRABGAP, RAB10, and DAPI; The enlarged figure is shown in the right upper corner. The arrow indicates the co-staining signals of MGCRABGAP, RAB10, and DAPI. Scale bar: 20 μm.

To determine whether MGCRABGAP interacts with RAB10, a co-IP assay, using NT2D1 cells, was preformed. First, lysates were immunoprecipitated with the IgG control or anti-FLAG antibody. Furthermore, these lysates were immunoblotted with the anti-FLAG antibody to confirm specific binding of the antibody (Figure 3A). Figure 3B indicates that the lysates showed strong signals through immunoblotting with the anti-RAB10 antibody (Figure 3B). Furthermore, to delineate whether MGCRABGAP colocalizes with RAB10 during mammalian spermatogenesis, an immunofluorescence assay (IFA) was performed on murine testicular sections. At stages X–XII of murine spermatogenesis, MGCRAGGAP was found to colocalize with RAB10 in elongating spermatids (Figure 4). These results indicated that MGCRABGAP interacts with and is colocalized with RAB10 in male germ cells. 

### 2.4. Dynamic Expression of RAB10 During Murine Spermatogenesis

**Figure 5:**
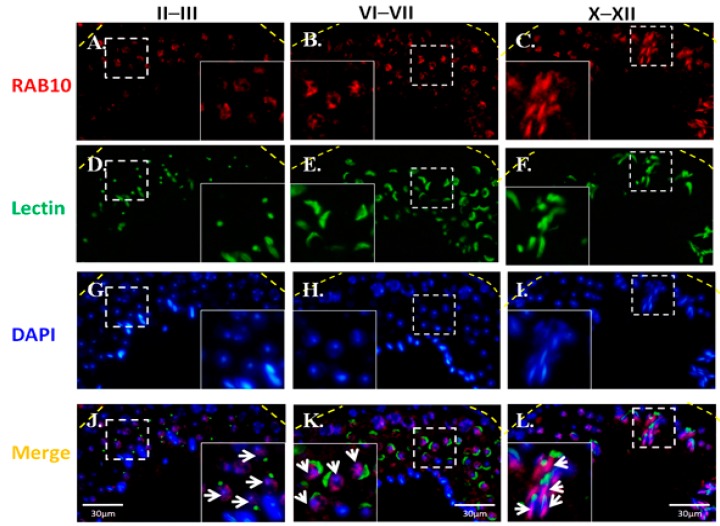
Expression patterns and localization of RBA10 during murine spermiogenesis; From left to right: stages II–III, stages VI–VII, and stage X–XII of spermatogenesis; (**A**–**C**), RAB10 signal (red); (**D**–**F**), Lectin (green, acrosome marker); (**G**–**I**), nuclear DNA (blue); (**J**–**L**), merge of RAB10, lectin, and nuclear DNA signals; The enlarged figures are shown in the right and left lower corners. The arrow indicates the RAB10 staining signals; DAPI: 4′,6-diamidino-2-phenylindole.

**Figure 6:**
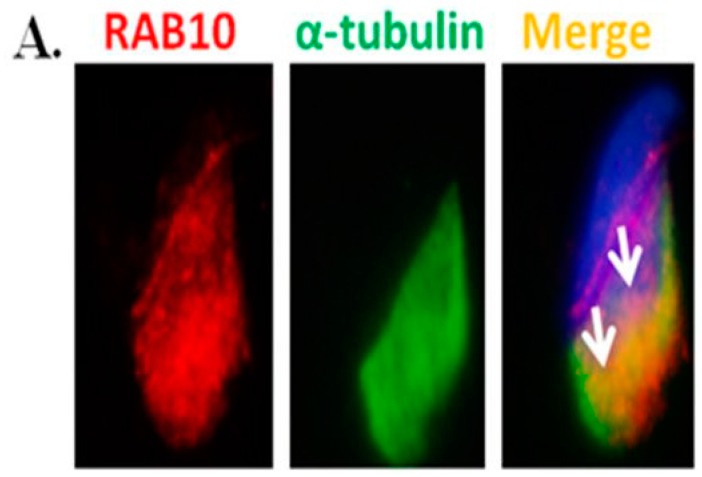

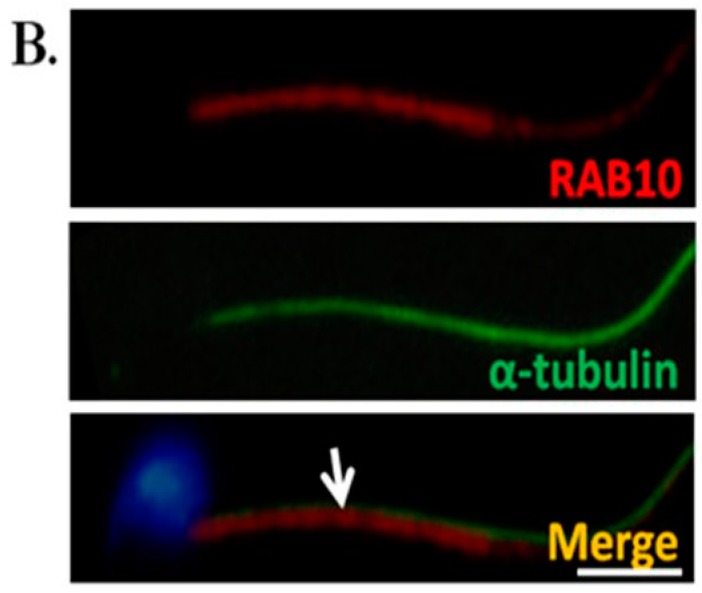
RAB10 is colocalized with α-tubulin at elongating spermatids and mature spermatozoa; Immunofluorescence detection of RAB10 and α-tubulin in elongating spermatids (**A**) and mature spermatozoa (**B**); The arrow indicates the co-staining signals of RAB10 and α-tubulin; Scale bar: 5 μm.

To reveal the localization of RAB during murine spermiogenesis, IFAs were performed on murine testis sections. RAB10 expression was initially observed in round spermatids at stages II–III during murine spermiogenesis (Figure 5A). Furthermore, RAB10 was localized around the sperm head of elongating spermatids (Figure 5B). At stages X–XII, RAB10 was expressed behind the acrosome region of elongated spermatids (Figure 5C). To determine the precise location of RAB10, testicular germ cell populations and spermatozoa were separated. In addition, α-tubulin was used as a marker of the manchette structure of the sperm head in elongating spermatids [37,38,39,40,41]. Figure 6A reveals that RAB10 and α-tubulin were colocalized in the manchette structure of elongating spermatids. In mature sperm, RAB10 was slightly expressed and colocalized with α-tubulin at the midpiece and the sperm tail (Figure 6B). These findings indicated that MGCRABGAP co-localized with RAB10 and was involved in sperm head and tail formation.

## 3. Discussion

In this study, we explored the possible interacting proteins of MGCRABGAP and discovered RAB10 as a critical interacting partner. Moreover, we determined the dynamic localization of MGCRABGAP and RAB10 complexes during mammalian spermiogenesis. This study, for the first time, identified possible interactors for MGCRABGAP in post-meiotic male germ cells.

### 3.1. GTPase-Activating Bioactivity of MGCRABGAP

GAPs activate the GTPases domain of RABs to enable transition from the GTP-bound active form to the GDP-bound inactive form [18]. GAPs can be categorized into at least five groups on the basis of their different structures: RHOGAPs, RASGAPs, RANGAPs, RABGAPs, and ARFGAPs [42]. MGCRABGAP is characterized by the presence of the TBC (Tre2/Bub2/Cdc16) domains; RABGAPs specifically activate RABs [17]. However, the actual enzymatic function of the GAPase-activating domain of MGCRABGAP has yet to be evaluated [16]. In this study, MGCCRABGAP was demonstrated to exhibit GAP bioactivity, as determined via the GTPase-activating assay. 

### 3.2. Cellular Functions of RAB10

RAB10 is widely distributed in many organelles such as the endoplasmic reticulum (ER), Golgi complex, and early and recycling endosomes; RAB10 mediates membrane trafficking within the cell and has extensive functions in cellular biological processes [43,44]. During axon development, RAB10 mediates membrane targeting of plasmalemmal precursor vesicles [34]. Furthermore, RAB10 has been indicated as a critical transport molecule in insulin-stimulated GLUT4-mediated recycling of adipocytes and basolateral recycling of MDCK cells [8,45,46]. In a knockout mouse model, the knockout of RAB10 disrupted the development of early mammalian embryos [47]. Because of its vital roles in cellular functions and very little known about RAB10 during mammalian spermatogenesis or spermiogenesis, we selected RAB10 for further analysis.

### 3.3. Possible Roles of RAB10 in Spermiogenesis

In this study, Figure 3A,B reveals that MGCRABGAP interacts with RAB10 as determined via co-IP in the NT2D1 cell line. Figure 4 also indicates that MGCRABGAP is colocalized with RAB10 in murine testicular sections, and RAB10 starts to be expressed at stage VI of spermatids (Figure 5). To further evaluate the roles of RAB10, the testicular germ cells were separated. Figure 6 indicates that RAB10 specifically colocalizes with α-tubulin, a specific manchette marker. The manchette structure is critical for the elongation and condensation of the spermatid nucleus and for the growth of the centrosome-divided axoneme [39,48,49]. This critical structure is formed in step 8 and disappears in step 15 of spermiogenesis. During the shaping of the sperm head, the manchette structure provides a scaffold for transporting cargos (e.g., proteins and cytosol) through microtubulins. Knockout of a critical manchette gene, *Hook1*, leads to a nonfunctional protein and results in an “abnormal spermatozoon head shape” (azh) murine strain [50,51]. Until now, only RAB27A/B has been suggested to be important in the transport of cargo on the manchette structure [52,53]. In our study, we found that RAB10 is also expressed in the manchette structure and may facilitate this process. In mature spermatozoa, according our results, RAB10 is localized at the peri-axonemal structure of mature spermatozoa. There are two possibilities for this: (i) RAB10 localizes at the peri-axonemal structure and is not present in the axoneme of the flagella or (ii) RAB10 localizes at the peri-axonemal structure and the axoneme of flagella. This study is the first to determine that the novel RAB, RAB10, and its regulator, MGCRABGAP, are co-expressed during the shaping of the sperm head and elongation of the sperm tail.

## 4. Experimental Section

### 4.1. Cloning, Transfection, and In Vitro GTPase-Activating Assay

For the synthesis of complementary DNA (cDNA) from a human RNA panel (Clontech, Mountain View, CA, USA), 13 µL aliquots of master mix containing 500 ng of human testis RNA, 1 µL of 500 ng/µL oligo(dT)_12–18_ primer (Invitrogen, Carlsbad, CA, USA), and distilled water were heated to 65 °C for 5 min and put on ice. Afterwards, 4 µL of 5× first strand synthesis buffer, 0.1 M dithiothreitol, 10 mM of each nucleoside triphosphate (dNTP), and 200 units of Superscript™ III Reverse Transcriptase (Invitrogen) were added for reverse transcription (RT) reactions. The RT temperature profile used was 42 °C for 1 h, 75 °C for 15 min, and final cooling to 4 °C. Aliquots of cDNA were stored at −20 °C until further use. Full-length *MGCRABGAP* transcripts were amplified using specific primers with engineered restriction enzyme sites (Forward: 5′-GGAATTCC-ATGACCACCCTCTCTCCTGA-3′; Reverse: 5′-GGGGTACCCC-TCAGAGGAAGAAATCCTTTAATG-3′), digested, and purified. Finally, ligation and transformation was performed using T4 DNA ligase (M0202; New England Biolabs, Ipswich, MA, USA) and JM109 competent cells, respectively (L2001; Promega, Fitchburg, WI, USA) [15,16]. The construct was confirmed via sequencing. Subsequently, NTERA-2 cl.D1 (NT2D1) cells (ATCC, Manassas, VA, USA), a pluripotent human testicular embryonal carcinoma cell line, were transfected with plasmids using Lipofectamine 2000 (Cat No.: 11668; Invitrogen, Carlsbad, CA, USA). Total cell lysates were collected to assess GTPase-activating bioability via an in vitro GTPase-activating assay and co-IP. The GTPase bioactivity of MGCRABGAP was evaluated using the RhoGTPase assay kit (Cat No.: BK105; Cytoskeleton, Denver, CO, USA). All steps were performed according to the manufacturer’s protocol.

### 4.2. Co-IP

To test whether MGCRABGAP interacts with RAB10, co-IP assay of NT2D1 cells was conducted. co-IP was performed according to our previous study [54]. The pFLAG-MGCRABGAP plasmid was transfected into NT2D1 cells using Lipofectamine 2000 (Invitrogen). Cell lysates containing 4 mg of protein in 1 mL was precleared by incubation with 50 µL of protein A/G beads (Santa Cruz Biotechnology, Santa Cruz, CA, USA) for 1 h at 4 °C on a rotator (15 rpm). Then, clarified supernatants were collected via centrifuging lysates at 1000× *g* for 30 s at 4 °C, and the supernatants were incubated overnight with either the control IgG or an anti-FLAG antibody (Cat No.: A5441; Sigma, St. Louis, MO, USA) at 4 °C on a rotator (15 rpm). Following, the samples were centrifuged (1000× *g* for 1 h at 4 °C). The immunoprecipitated samples were collected and then washed twice with 1× phosphate-buffered saline (PBS). Thereafter, immunoblotting was performed using a primary anti-RAB10 antibody (Cat No.: 8127; Cell Signaling Technology, Boston, MA, USA) and a secondary antibody, and then, the immunoblots were exposed using X-films (Thermo Fisher Scientific, MA, USA). 

### 4.3. MS Analysis

Immunoprecipitated protein mixtures were reduced with dithiothreitol, *S*-alkylated with iodoacetamide, and digested with Lys-C and trypsin, as previously described [55]. The digested peptides were desalted using SDB-XC StageTip (3M Company, St. Paul, MN, USA), followed by SCX StageTip (3M Company) [27], and were analyzed using nano liquid chromatography-mass spectrometry/mass spectrometry (LC-MS/MS) on the Dionex Ultimate 3000 RSLC nano system (Thermo Fisher Scientific, Waltham, MA, USA) with an LTQ Orbitrap XL mass spectrometer (Thermo Fisher Scientific). Protein identification was performed as described in our previous study [56]. 

### 4.4. Separation of Testicular Germ-Cell Populations

The animal studies were approved by the Institutional Animal Care and Use Committee of Fu Jen Catholic University (A10180). Testes were obtained from adult mice (C57BL/6, *n* = 2, postnatal days > 80). Spermatogenic cells were separated using a centrifugal system based on the density of different types of germ cells as modified from a previous protocol [57,58]. After de-capsulation of testes, seminiferous tubules were digested in DMEM/F12 media with trypsin (1 mg/mL), collagenase (0.75 mg/mL), DNAase I (5 μg/mL), protease inhibitor cocktail (1×, Sigma-Aldrich, Shanghai, China), and antibiotics (1×, Invitrogen). The mixture was incubated within 140 cycles per min for 1.5 h at 37 °C. Germ cells suspensions were filtered through 35 μM nylon filters (Falcon; Becton Dickinson, Franklin Lakes, NJ, USA) and followed by centrifugation using a Kubota centrifuge 3330 (Kubota Corp., Tokyo, Japan). Four suspension solutions were collected after centrifuging at 700× *g*, 400× *g*, 200× *g*, and 100× *g*. Finally, the cell pellets were collected from these suspensions after centrifuging at 3000× *g*. Pellets were suspended in 1× PBS and spread on a slide and air-dried for downstream immunofluorescence assays. Mature spermatozoa were collected from the cauda epididymis of adult male mice.

### 4.5. Immunofluorescence Assay

For immunofluorescence assays (IFAs), the slides were treated with 0.1% Triton X-100; washed twice with Tris-buffered saline (TBS); and then followed by incubation with anti-MGCRABGAP (Cat No.: ab89799; Abcam, Cambridge, UK), anti-Rab10 (Cat No.: ab104859; Abcam), and a monoclonal antibody against α-tubulin (Cat No.: ab52866; Abcam) for 60 min at room temperature. After washing with TBS, the sections were incubated with goat anti-rabbit Alexa Flour 488 and goat anti-mouse Alexa Flour 568 (Molecular Probes, Carlsbad, CA, USA) for 60 min at room temperature and then washed again with TBS. Lectin (Lectin) peanut agglutinin conjugated to Alexa Fluor 568 (Molecular Probes, Carlsbad, CA, USA) was used to locate the acrosome of male germ cells. Moreover, 4′,6-diamidino-2-phenylindole (DAPI) was used to stain nuclei.

### 4.6. Data Analysis

GTPase-activating assays were performed in quadruplicate and statistically accessed via paired *t*-test methods. * indicates *p* < 0.05, which was considered statistically significance. The developmental stage of spermatids from IFAs were classified according to the staining patterns of acrosomal morphology using the acrosomal marker lectin [59].

## 5. Conclusions

Based on the presented evidence, we propose that MGCRABGAP is involved in sperm head and tail formation of post-meiotic male germ cells by regulating its interactor RAB10.

## Figures and Tables

**Table 1:** Identification of possible interactors of MGCRABGAP through coimmunoprecipitation (co-IP) and nano liquid chromatography-mass spectrometry/mass spectrometry (nano LC-MS/MS).

Symbol	Localizations/Functions
RAB10	Localized to exocytic and endocytic compartments/Regulates intracellular vesicle trafficking and GLUT4 [30,32]
RAB5C	Localized to early endosomes/Regulates the kinetics of membrane traffic in the early endocytic pathway and modulates Rac-mediated cell motility [28,29]
RAB1A	Localized to Golgi/endoplasmic reticulum trafficking/Regulates early endocytic vesicle sorting [33]
RAP1A	Localized to the cell membrane and cytosolic region/Regulates ERK activation and integrin-mediated cellular functions [34,35,36]
RAP1B	Similar to RAP1A.
